# Unclogging the effects of the Angiojet® thrombectomy system on kidney function: a case report

**DOI:** 10.1186/s13256-021-03062-3

**Published:** 2021-09-10

**Authors:** Tayeba Roper, Muhammad Amaran, Prakash Saha, Cormac Breen, David Game

**Affiliations:** 1 Department of Renal Medicine, Guy’s & St Thomas’ NHS Foundation Trust,, Great Maze Pond, London, SE1 9RT United Kingdom; 2 Vascular Surgery Department, Guy’s & St Thomas’ NHS Foundation Trust, St Thomas’ Hospital, London, United Kingdom

**Keywords:** Acute kidney injury, Haemolysis, Deep vein thromboses, Arterial thromboses, Angiojet

## Abstract

**Background:**

AngioJet® is an increasingly used method of percutaneous mechanical thrombectomy for the treatment of patients with arterial and venous thromboses. AngioJet® has been shown to cause intravascular haemoylsis universally. We report the case of a 29 year old patient who underwent AngioJet® thrombectomy and post-procedure developed a stage 3 Acute kidney injury (AKI.) requiring renal replacement therapy (RRT), secondary to intravascular haemolysis. We aim to explore the mechanism and potential risk factors associated with developing AKI in these patients and suggest steps to optimise patient management.

**Case presentation:**

A 29 year old Caucasian male who developed a stage 3 AKI, requiring RRT, following AngioJet® thrombectomy for an occluded femoral vein stent. Urine and laboratory investigations showed evidence of intravascular haemolysis, which was the likely cause of AKI. Following a brief period of RRT he completely recovered renal function.

**Conclusions:**

AKI is an increasingly recognised complication following AngioJet® thrombectomy, but remains underappreciated in clinical practice. AKI results from intravascular haemolysis caused by the device. Up to 13% of patients require RRT, but overall short-term prognosis is good. Pre-procedural risk factors for the development of AKI include recent major surgery. Sodium bicarbonate should be administered to those who develop renal impairment. Renal biopsy is high risk and does not add to management. Increased clinician awareness and vigilance for AKI post-procedure can allow for early recognition and referral to nephrology services for ongoing management.

## Background

Arterial and deep venous thromboses (DVT) are common and can cause significant morbidity and mortality. The mainstay of treatment most commonly involves the administration of antiplatelet or anticoagulant medications, respectively. However, for larger burden clots more invasive treatment options are available, to reduce the associated risk of complications, including clot embolisation and post thrombotic syndrome (PTS). Traditional methods for clot removal with catheter directed thrombolysis (CDT) are now being superseded by Percutaneous mechanical thrombectomy (PMT) devices, such as the AngioJet® rheolytic thrombectomy device (Possis Medical, Minneapolis, Minnesota, USA) (henceforth AngioJet®). These are an increasingly used form of endovascular treatment for both arterial and deep vein thromboses, due to the associated reduction in treatment time, intensive care admissions, and overall length of hospital stay compared to CDT techniques [1, 2].

Angiojet® employs multiple high-pressure saline jets which cause fragmentation of targeted clots, whilst simultaneously delivering thrombolytic agent into the clot. A Venturi effect is created by the jets, which allows for aspiration of the clot debris, and prevents clot embolisation [3]. Although effective, the mechanism of action has been shown to cause significant haemolysis and routinely results in post-procedural haemoglobinuria. This in turn can cause acute kidney injury (AKI), which although an increasingly recognized complication of Angiojet®, remains underappreciated in clinical practice. Five previous cases of AKI following Angiojet® have been reported in the literature, one of which was in a child [4–8]. We report the case of a 29 year old male who developed a severe Stage 3 AKI [9], requiring renal replacement therapy (RRT), following AngioJet® thrombectomy of an occluded iliac vein stent. We aim to expand on the possible risk factors for development of AKI in patients undergoing AngioJet®, and suggest steps which could be taken to optimise the management of these patients.

## Case presentation

**Fig. 1:**
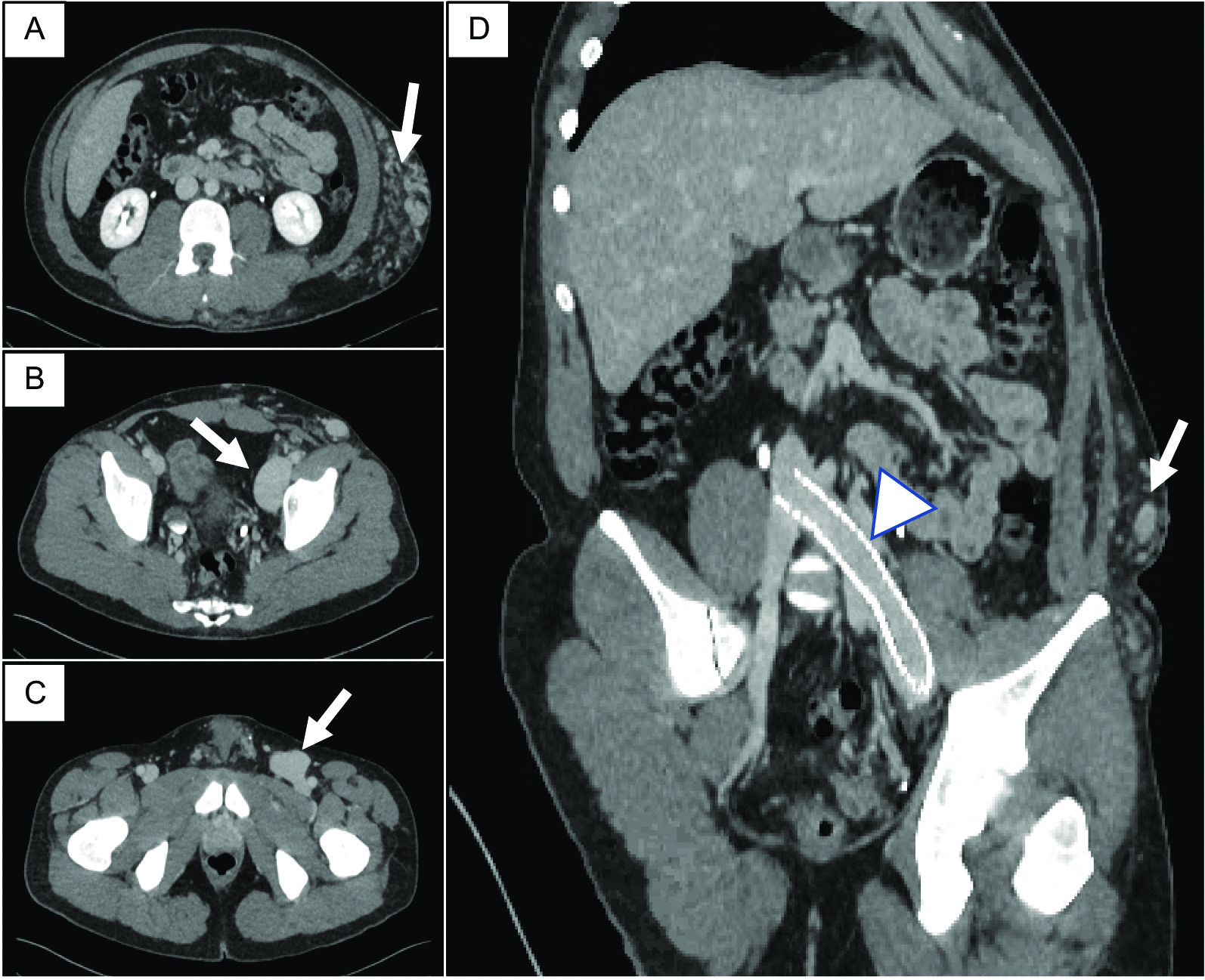
**A**–**C** Computed tomography with contrast agent showing the vascular malformation (arrow) and placement of venous stent (**D**, arrow head)

**Fig. 2:**
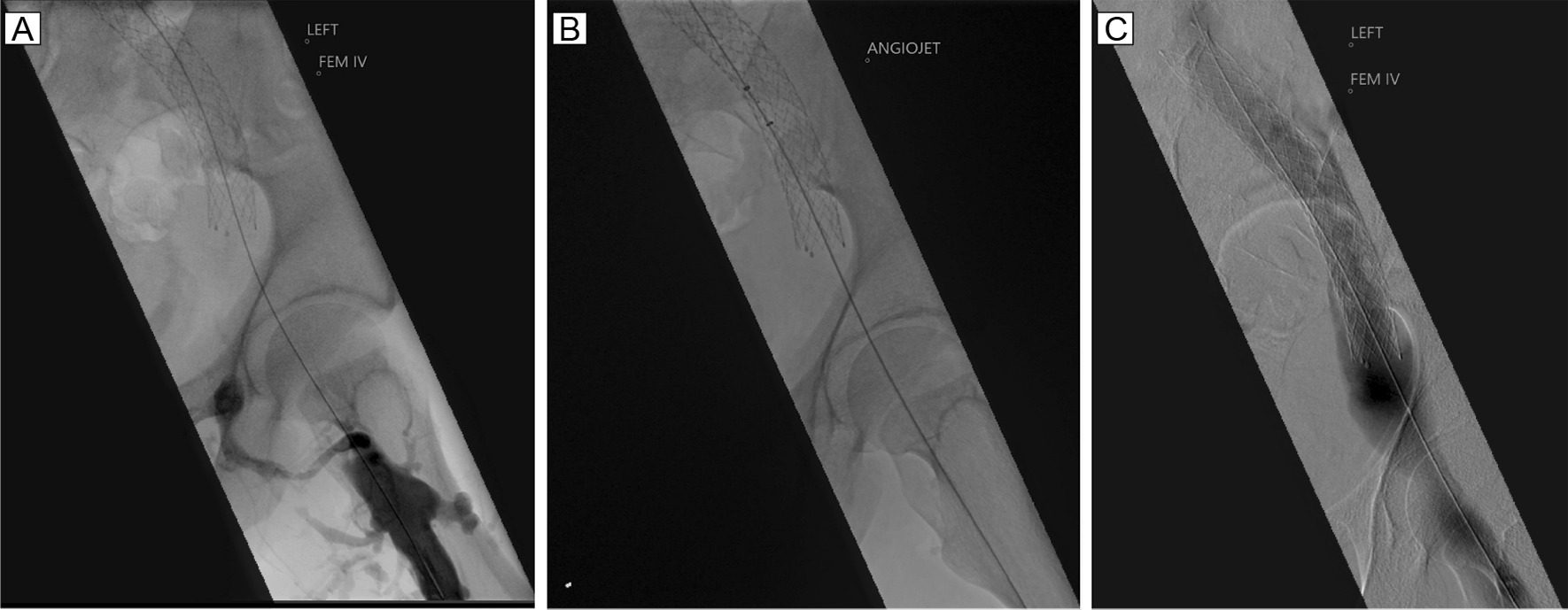
Venogram demonstrating occluded stent (**A**), Angiojet thrombectomy (**B**) and successful recanalization of the stent (**C**)

A 29 year old Caucasian male with a known left flank symptomatic venous malformation (VM) (Fig. 1) was admitted with a 2-day history of left leg pain, swelling and discolouration secondary to DVT. There was no history of chest pain, shortness of breath or palpitations. A year prior he had undergone left common-iliac vein stenting for a non-thrombotic iliac vein lesion, to redirect venous return away from the VM. As he remained symptomatic following this procedure, elective surgical excision-and-tie of the main feeder vessel to the VM was performed three weeks prior to this presentation. Bleeding at the time of this operation led to Apixaban, that he was previously on, to be stopped. He had no other past medical history, including no known history of renal impairment, and no family history of renal disease. At the time of presentation cardiorespiratory examination was unremarkable. Examination of the abdomen revealed a firm, palpable mass in the left abdominal wall, consistent with the known VM. The left upper leg was swollen with mottling of the skin, but otherwise soft and non-tender, and peripheral pulses were intact. 7500 units twice daily of low molecular weight heparin (LMWH) were commenced at the time of presentation. Following CT venography (Fig. 1) and duplex ultrasonography, that identified an occluded venous stent, Angiojet thrombectomy and venoplasty were performed under general anaesthetic by the vascular surgical team (Fig. 2). Pre-operative clotting markers were all within normal limits (INR 1.1, APTR 1.1). Intraoperatively, 8000 units of unfractionated heparin were administered, followed by 15000 units of LMWH one hour post-procedure. Successful recanalisation of the thrombosed stent was achieved. In the post-operative period he developed bradycardia and vomiting, and was treated with antiemetic and intravenous fluids. Vomiting settled after 36 hours. He remained haemodynamically stable throughout. Following surgical intervention a continuous intravenous heparin infusion was commenced, to prevent re-occlusion of the stent.

**Fig. 3:**
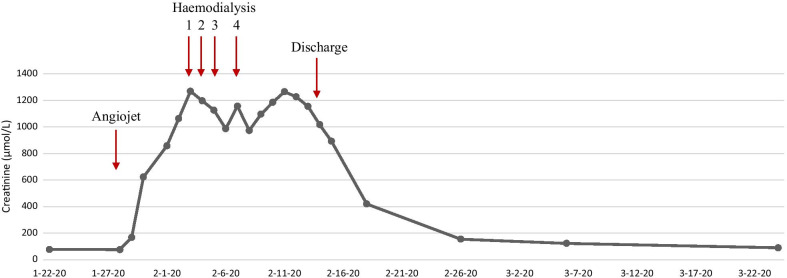
Graph of Creatinine over time. Arrows demonstrate timing of Angiojet® thrombectomy, Haemodialysis sessions and discharge

His renal function was noted to decline immediately postop, from a baseline serum creatinine of 77 µmol/L to 168 µmol/L (Fig. 3). The patient passed dark red urine, which on urine dipstick tested positive for blood. Renal function continued to decline over the coming 48 hours (Fig. 3). Laboratory investigations demonstrated serum lactate dehydrogenase (LDH) to be elevated at 1148 U/L and haptoglobin level low at 0.3 g/L, haemoglobin fell post-procedure from 145 to 86 g/L (Table 1). Direct antiglobulin test was negative. Blood tests performed pre-procedure and within 72 hours post-procedure are shown in Table 1. Acute renal screen blood tests and virology were all negative. An ultrasound of the kidneys and urinary tract demonstrated normal sized (right 12.5 cm, left 11.9 cm), unobstructed kidneys bilaterally, with a diffuse increase in renal echogenicity and loss of corticomedullary differentiation. Incidentally the spleen was noted to be enlarged at 13 cm. A duplex ultrasound confirmed patent renal vasculature, and good perfusion of both kidneys.

**Table 1:** Laboratory investigations pre- and within 72 hours post-Angiojet® thrombectomy

Laboratory investigation	Pre-procedure	Within 72 hours post-procedure	Reference range
Haemoglobin	145	86	130–170 g/L
White blood cells	9.3	7.1	4–11 × 10^9^
Platelets	196	191	150–400 × 10^9^
Creatinine	77	1296	59–104 µmol/L
Estimated glomerular filtration rate	104	4	70–130 mL/min
Urea	–	28.8	1.7–8.3 mmol/L
Potassium	4.3	4.9	3.5–5.0 mmol/L
Sodium	138	138	135–145 mmol/L
Bicarbonate	–	16	22–30 mmol/L
Albumin	52	38	40–52 g/L
Bilirubin	7	5	0–21 umol/L
Creatinine kinase	–	201	0–229 IU/L
C-reactive protein	1	31	0–4 mg/L
International normalised ratio	1.1	1.0	0.8–1.2 Ratio
Activated partial thromboplastin time ratio	1.1	2.4 (on Heparin infusion)	0.8–1.2 Ratio
Haptoglobin	–	0.3	0.3–2.0 g/L
Reticulocyte count	–	66	10–100 × 10^9^
Lactate Dehydrogenase	–	1148	135–225 U/L
Direct Antiglobulin Test	–	Negative	–
Blood film	–	Red cell morphology—mild polychromasia, slight anisocytosis, rare tear drop poikilocytes	–

The patient was transferred to the renal ward 72 h post-procedure due to an ongoing decline in renal function and fall in urine output (Fig. 2). He was initially managed with intravenous 1.26% sodium bicarbonate and 0.9% sodium chloride solutions, to maintain a positive fluid balance. However urine output continued to fall and he started to develop evidence of fluid overload. After a further 48 hours, intermittent haemodialysis (HD) was commenced via a right internal jugular vein vascath. Four sessions of HD were completed in total (Fig. 3). He subsequently started to show signs of renal recovery with polyuria, passing over 3 litre of clear urine per day. A decision was made not to perform a renal biopsy in view of the high risk of bleeding given the concomitant heparin infusion. He was discharged with a falling creatinine and once loaded on warfarin. At the time of writing the patient’s renal function had improved to near baseline, with a serum creatinine of 90 µmol/L (Fig. 3).

## Discussion and conclusions

Haemolysis is a well-documented cause of AKI in many conditions, including autoimmune haemolysis, paroxysmal nocturnal haemoglobinuria and haemolysis secondary to prosthetic cardiac valves [10]. AngioJet® has previously been shown to universally result in post-procedural gross haematuria, following intravascular haemolysis caused by the high-pressure saline jets [11]. Furthermore, previous cases of AKI secondary to AngioJet®-induced intravascular haemolysis have also been reported [4–8]. The occurrence of haemolysis in the case presented, as evidenced by the passage of dark red urine post-procedure, fall in haemoglobin and haptoglobin, and rise in serum LDH, was an anticipated consequence of the procedure. Given the patients’ young age and absence of other risk factors, deterioration in renal function to the point of requiring RRT (Fig. 3), was not anticipated. The patient had a small volume of contrast intra-operatively and significant vomiting post-operatively, both of which could have contributed to AKI. The severity of AKI with need for RRT, despite aggressive fluid replacement, suggests the cause of deterioration in renal function was likely haemolysis, as previously reported.

Previous reports have demonstrated an increased risk of complications following native renal biopsy in hospital in-patients who develop AKI, compared to outpatients [12]. Given this and the concomitant Heparin infusion, our patient was started on post AngioJet®, the decision was made not to perform a renal biopsy to further investigate the cause for AKI. It was felt that there was sufficient evidence of haemolysis (as previously discussed) as a cause for AKI, and that a biopsy would add little to guide further management. One previous study reports on renal biopsy findings in a patient who developed AKI post AngioJet®. This study reported findings including acute tubular injury, red blood cell debris within tubules, and tubular epithelial cells and podocytes staining for ferritin and haemo-oxygenase-1 (HO-1) [7]. These findings lend support to numerous studies which suggest the mechanism of AKI following haemolysis is likely related to a complex interplay of cytotoxic inflammatory mediators, activated in response to the increased iron and haemprotein load from lysed red blood cells. Filtered haemoproteins induce the release of ferritin and HO-1, which protect against oxidative-stress by scavenging free haem and iron. When these protective mechanisms are overwhelmed however, haem and iron can have direct toxic effects on glomeruli and tubular cells, resulting in renal dysfunction [13].

The ‘Peripheral Use of AngioJet Rheolytic Thrombectomy with a Variety of Catheter Lengths’ (PEARL) registry only briefly mention the association between AngioJet® and the development of AKI. PEARL made no comment on the incidence of AKI not requiring RRT, and quoted that 5% of patients required RRT at 12 months post-procedure. They did not however expand on the indication for RRT, nor resolution and prevention of AKI in this group [14]. Subsequent studies have reported on the risk of AKI associated with AngioJet®. Morrow *et al*. observed the incidence of AKI in patients with arterial and venous thromboses, undergoing PMT with AngioJet®. They found the incidence of renal dysfunction to be significantly higher in the PMT group compared to CDT controls, 21% and 0% (*p* = 0.033), respectively. None of the PMT patients however required RRT [15]. Similarly, Escobar *et al*. found AngioJet® to be an independent risk factor for the development of AKI (odds ratio 8.22, *p* = 0.004) [16]. Shen *et al*. also reported a significantly increased risk of AKI in patients undergoing AngioJet® for iliofemoral DVT compared to CDT, 22.8% and 9.2% (*p* = 0.013), respectively. Furthermore, they demonstrated major surgery within 3 months prior to vascular intervention, to be a risk factor for the development in AKI post AngioJet® (odds ratio 8.51, *p* < 0.01) [11]. Our patient underwent excision-and-tie of the VM within 3 months prior to AngioJet®, potentially placing him at increased risk of developing AKI. Other than major surgery performed within 3 months of vascular intervention [11], none of the studies identified any pre-procedural risk factors for the development of AKI, including traditional risk factors for AKI. Both Escobar *et al*. and Shen *et al*. reported 2 patients requiring a period of RRT, 11% and 13%, respectively [11, 16].

The case presented appears typical when compared to other reported cases of significant AKI following AngioJet® [4–8]. Our patient developed AKI immediately post-procedure with associated haematuria and evidence of haemolysis, despite aggressive intravenous rehydration. After a brief period of HD there was evidence of renal recovery with increased urine output and improvement in serum creatinine (Fig. 3). It remains unclear as to whether the presence of a VM contributed to the development of AKI in our patient. The presence of a VM meant there was a larger burden of thrombus present, which in turn would require a more prolonged procedure to clear. It is conceivable that the increased clot burden allowed for a greater degree of haemolysis and therefore an increased risk of AKI in this patient. Prevention and management of AKI associated with haemolysis is an area which remains under investigation. There is some evidence to suggest the use of sodium bicarbonate may be beneficial through the effect of alkalinisation, reduction of free radical generation and attenuation of the effects of oxidative stress on renal tubules [13] (Table 2). In some individuals however, these conservative measures are unsuccessful and the need for RRT may be inevitable. Timely referral to nephrology services allows for advice regarding fluid resuscitation and commencement of RRT, potentially without the need for admission to an intensive care unit. This case, along with previous reports, would suggest that the short-term prognosis in patients who develop AKI post AngioJet® is good, with good recovery of renal function in most. Further studies are needed however to determine the potential long-term implications of AKI following AngioJet®, including the long-term risk of needing RRT.

**Table 2:** Management pre- and post- AngioJet® for optimization of patient care

Management pre- and post- AngioJet® thrombectomy
Patients should be counselled and consented regarding the potential risk of AKI and need for RRT
Consider use of alternative therapy in patients who have undergone major surgery within the last 3 months
Renal function should be checked immediately post-procedure
Fluid resuscitation with intravenous sodium bicarbonate, should be initiated immediately in those with renal impairment
An accurate fluid balance chart should be maintained
Timely referral to nephrology services will allow for appropriate commencement of renal replacement therapy and avoidance of ICU admission
Performing a renal biopsy is high risk and does not alter management in those with renal impairment

AKI is an increasingly reported complication following AngioJet® thrombectomy, but remains underappreciated in day-to-day clinical practice. AKI can be severe and in up to 13% of cases require RRT, but short-term outcomes are good. Routine risk factors for the development of AKI in hospital inpatients, are not associated with AngioJet®. Undergoing major surgery within 3 months of AngioJet® is the only pre-procedural risk factor reported to be associated with the development of AKI. The use of CDT over AngioJet® may therefore need to be considered in these potentially at risk patients. Measures to prevent AKI following haemolysis remain under investigation, however the administration of sodium bicarbonate may be beneficial. Performing a renal biopsy to investigate these patients is high risk and we feel does not offer any clinical benefit. Clinicians should be mindful of the risk of AKI associated with AngioJet® thrombectomy in order to allow for; appropriate counselling and consent pre-procedure; post-procedural vigilance for deterioration in renal function; and timely referral to nephrology services in the event of developing AKI (Table 2).

## Data Availability

Not applicable.

## Abbreviations

AKI: Acute kidney injury
CDT: Catheter directed thrombolysis
DVT: Deep vein thromboses
HD: Haemodialysis
LDH: Lactate dehydrogenase
LMWH: Low molecular weight heparin
PEARL: Peripheral Use of AngioJet Rheolytic Thrombectomy with a Variety of Catheter Lengths
PTS: Post thrombotic syndrome
RRT: Renal replacement therapy
VM: Venous melaformation
